# Hereditary spastic paraplegia associated with a rare *IFIH1* mutation: a case report and literature review

**DOI:** 10.1186/s41065-019-0104-x

**Published:** 2019-08-13

**Authors:** Nan Liu, Jiajun Chen, Chuan Xu, Tianji Shi, Jia Li

**Affiliations:** Department of Neurology, China-Japan Union Hospital of Jilin University, Changchun, Jilin Province People’s Republic of China

**Keywords:** Hereditary spastic paraplegia, *IFIH1*, Missense mutation

## Abstract

Here, the pathogenesis of an *IFIH1* gene mutation is discussed through the analysis of a sporadic case of hereditary spastic paraplegia. Next-generation sequencing was performed for the patient and his family members to detect mutations at the *IFIH1* locus. The patient and his father were found to carry the same heterozygous missense mutation (c.1093A > G; p.Gly495Arg), while the patient’s mother does not carry this mutation. This is the first report of this heterozygous *IFIH1* mutation and it is predicted to be disease-causing.

## Introduction

Hereditary spastic paraplegia (HSP) is a rarely observed neurodegenerative disease. The main pathological feature of HSP is corticospinal tract degeneration, which is clinically reflected as myasthenia and progressive spasms of the lower limbs. A case of sporadic hereditary spastic paraplegia was admitted into our hospital. Based on the results of gene testing using an HSP gene panel, the disease-causing gene was determined to be *IFIH1* (c.1093A > G). Hereditary spastic paraplegia related to *IFIH1* gene mutations is very rarely observed in practice. In this paper, this case is described and the clinical features, types, and characteristics of the various disease-causing gene mutations causing HSP have been summarized based on the relevant literature. The ethics committee of China-Japan Union Hospital of Jilin University approved this study.

### Patient data

The patient is a 35-year-old male. He visited the outpatient department of the hospital in July 2017 because of stiffness in his left and right lower limbs that had persisted for the past 3 years and 1 year, respectively. Three years ago (2014), the patient felt stiffness in his left lower limb when running but did not consult a physician. This stiffness progressively worsened as reflected by stiffness being felt even when walking on flat ground and the development of an abnormal gait. His symptoms were more severe when he felt cold or anxious and were improved by movement. One year ago (2016), he began to feel stiffness and reduced flexibility in his right lower limb, which was accompanied by paroxysmal muscle spasms. He denied any muscular pain and the spasms abated after several seconds. In addition, he denied any disturbance of consciousness and reported that his upper limbs were functioning normally. When visiting the hospital in 2017, the patient was suffering from stiffness in both lower limbs and could not walk. The patient’s medical history as follows: one year ago, the patient received surgery at another hospital for lumbar disc herniation. He denied any decrease in cognitive function or having any immunological diseases such as rashes, photosensitivity, oral ulcers or swollen joints. He also denied any history of infectious diseases such as hepatitis, tuberculosis, typhoid fever or malaria. The patient had normal growth and development during childhood and had normal athletic ability.

### Clinical report

The patient presented with a normal mental state and could express his opinions clearly. It was determined through rough measurement that he had an above average intelligence and normal functioning of cranial nerve I. He showed no sign of general muscular atrophy and had normal muscle tension in his upper limbs. Obviously increased tension in his lower limbs was observed, and the muscle strength of his four limbs was determined to be at Level V. He had a spastic gait, and active reflexes of his lower limbs and tendons were observed. The bilateral pathological conditions of the patient were determined to be (+).

### Auxiliary examinations

The patient had an EMG after visiting a hospital in February 2016. His head MRI showed no obvious abnormality. Cervicothoracic junction MRI showed no abnormal intramedullary signals or diminution of the spinal cord. He underwent a routine blood examination, four infectious disease tests, a blood coagulation test, a hepatic-renal function examination, and routine urine and stool tests in November 2016, none of which showed any obvious abnormalities. In addition, he also had an immunity-related ESR, ANA, ENA, AQP-4, Ig test, complement test, anti-GAD antibody test, six immunohistochemical tests (−), a tumor marker test (−), a routine lumbar puncture, and a cytological examination, none of which showed any obvious abnormalities.

### DNA sequencing

DNA sequencing for the patient and his family members was conducted using a next-generation sequencing platform. MutationTaster (http://www.mutationtaster.org) and the Human Gene Mutation Database were applied to predict the pathogenicity of the identified genetic variant.

## Results

**Fig. 1:**
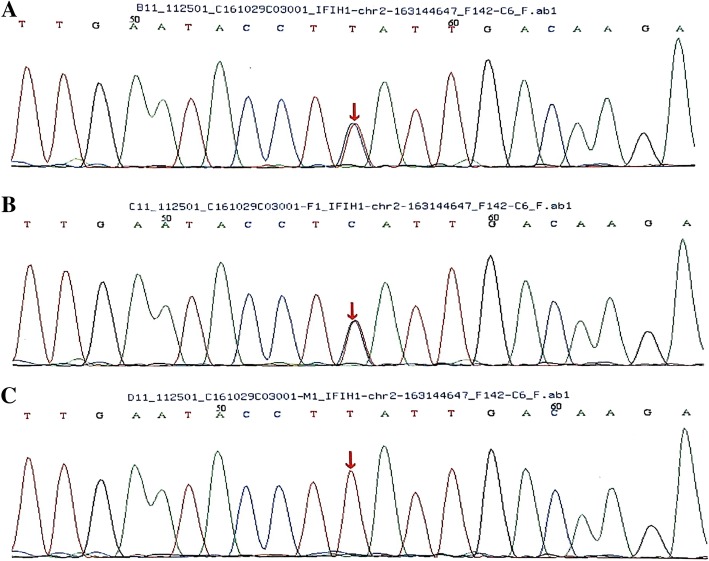
Sequence analysis of the *IFIH1* gene. DNA samples were provided for sequencing by the patient and his parents. **a** The patient carries a heterozygous c.1093A > G variant resulting in the missense mutation, p.K365E. **b**The patient’s father also carries a heterozygous mutation at this site. **c** The patient’s mother has no mutation at this site

Next-generation gene sequencing based on the HSP test kit indicated the presence of one heterozygous mutation in the *IFIH1* gene in the patient: c.1093A > G (Fig. 1a). His father carries the same heterozygous mutation (Fig. 1b), while his mother does not carry a mutation at this site (Fig. 1c). According to MutationTaster, this mutation results in the amino acid substitution, p.K365E (missense mutation). This mutation does not occur at a polymorphic site; thus, the frequency of its occurrence is likely extremely low. This mutation was not present in the Human Gene Mutation Database and has not been previously published.

## Discussion

Hereditary spastic paraplegia is also known as Strumpell-Lorrain disease. According to traditional classification, this disease can be divided into the simple type of HSP (clinical manifestations include typical muscle spasms, hyperreflexia, clonus, gait disorder, and bladder dysfunction) and the complex type, where, in addition to the above clinical manifestations, the upper limbs may be affected and there may be optic nerve atrophy, retinal decolorization, cataracts, cerebellar dysfunction, cognitive dysfunction, peripheral neuropathy, epilepsy, deafness, and ichthyosis [1, 2]. These symptoms are caused by degeneration or injury related to distal axonopathy along the corticospinal tract.

The pathogenesis of HSP remains uncertain, and the axonal degeneration caused by the various types of HSP differ in their underlying molecular pathogenesis. Known disease-causing genes associated with HSP mainly encode proteins involved in the morphology of the endoplasmic reticulum, microtubule dynamics and transport, mitochondrial function, lipid metabolism, and endosome/lysosome functions. Study has indicated that HSP may also be caused by defects in transport of proteins,, lipids, and other substances through the cell [3]. The main pathological change caused by HSP is axonal degeneration, which may also be accompanied by other changes such as demyelination and neuron loss. Through autopsy, axonal degeneration of the corticospinal tract (most obvious in the thoracic spinal cord) and fasciculus gracilis fibrosis (most obvious in the cervical spinal cord) have also been detected [1].

Early diagnosis is crucial for HSP patients. However, HSP is characterized by low incidence, latent progression, and heterogeneous symptoms, which make this disease clinically difficult to recognize and diagnose. In addition to the clinical manifestations, the identification of gene mutations serves as the basis for diagnosis but HSP is also considered to be one of the most genetically heterogeneous of the nervous system diseases. To date, 84 different loci and 67 disease-causing genes have been identified and included in genetic models of HSP. Inheritance modes of the disease include autosomal dominant inheritance (AD), autosomal recessive inheritance (AR), X-linked inheritance, and the rarely seen mitochondrial inheritance [4]. Modes of mutation include deletions, insertions, repeats, missense mutations, and splicing site mutation [5]. The incidence of HSP is 0.5–5.5/100,000 for AD and 0.3–5.3/100,000 for AR [6]. Although HSP is characterized by a very high degree of genetic heterogeneity, in 40–70% of all families with HSP there is no molecular diagnosis possible based on current knowledge. Thus, it is likely that a large number of disease-causing genes, pathogenetic mechanisms, and modes of inheritance remain unknown [7].

Heterozygous mutations of *IFIH1* (2q24.2) may cause HSP and that the pathomechanism is related to levels of Type I interferon. However, based on our extensive literature review, only one case of hereditary spastic paraplegia related to mutations of *IFIH1* has been reported [10]. The patient was a British male (Caucasian). Commonly seen clinical manifestations among patients in the UK include early-onset spastic paraplegia accompanied by multi-system inflammation, including interstitial lung diseases, oral ulcers, diffusive hair loss, dermatomyositis, Raynaud’s phenomenon. and arthritis [8, 9]. When the patient was 33 years old, he began to suffer obvious spasms in his lower limbs but his upper limbs were unaffected. MRI of the patient’s head and vertebral column showed no significant abnormalities. Exome sequencing indicated a missense IFIH1 mutation (c.1483G > A; p.Gly495Arg), which was reflected as spastic paraplegia that was correlated to the level of Type I interferon.

IFIH1 (human interferon induced with helicase C domain 1, also known as MDA5 or melanoma differentiation-associated protein 5), is a cytoplasmic virus RNA acceptor containing 1025 amino acids. In vitro functional analysis indicates that *IFIH1* mutations can increase the expression level of Type I interferon, and the transcription levels of interferon-induced genes are thereby also increased [11]. The neuroimmunological characteristics caused by heterozygous mutations of *IFIH1* are closely related to this increased level of interferon, which can result in activation of the immune system. The type I interferon system is a component of human antiviral immunity, and because type I interferon can exert a neurotoxic effect, improper stimulation may cause inflammatory diseases [8]. Indeed, some reports indicate that *IFIH1* mutations are related to various autoimmune diseases such as diabetes mellitus Type I, systemic lupus erythematosus, Graves’ disease, multiple sclerosis, rheumatoid arthritis, Hashimoto’s thyroiditis, and autoimmune Addison’s disease [12].

In the case reported here, only the patient and his parents underwent gene testing. The patient and his father share the same heterozygous mutation at the identified site, while his mother has no such mutation. This *IFIH1* mutation (c.1093A > G) has not been published elsewhere. The sequencing results indicated that this missense mutation is not a sequencing error; however, the patient’s father showed no clinical symptoms but this may be related to penetrance and the influence of genetic modifications and environmental factors [13]. The patient’s child has not undergone gene testing as he is still young and has no clinical symptoms. Moreover, due to certain limitations, other family members have not undergone gene testing, so the mode of inheritance is still uncertain.

At present, there are only a few options available for the treatment of spastic paraplegia. In addition to rehabilitation therapy and physical therapy for the maintenance of muscular strength and coordinated movement, certain medications such as oral baclofen, intramuscular injections of botulinum toxin or intrathecal injections of baclofen can relieve spasms to a degree [4]. Identification of the underlying molecular defects in individual cases of HSP is key to furthering the research and development of new treatment options to enable individualized therapy. Generally, HSP has no impact on the lifespan of patients but it can cause serious disability. Accurate genetic diagnosis, genetic counseling, and symptom management are crucial for the benefits these provide to patients with HSP and their families. In addition, identification of gene mutations among family members of HSP patients is beneficial for the early detection of non-symptomatic mutation carriers and the timely implementation of intervention measures to slow disease progression.

## Conclusion

Here, we identified a new disease-causing mutation of the *IFIH1* gene, c.1093A > G. To the best of our knowledge, there have been no previous reports on this gene mutation, whether in association with HSP or other diseases. Further investigations are necessary, including gene testing of the patient’s family members and animal and cell experiments to validate the effect of this mutation. As indicated in our report, molecular diagnosis is still a major challenge in the management of HSP. Besides the ever-increasing number of known HSP disease-causing genes, clinical prediction of disease evolution and prognosis remains complex in the face of heterogeneous disease phenotypes even among patients carrying the same mutation remains. Some patients experience early onset, some suffer serious symptoms, and some never shown any symptoms. These issues provide avenues for the future development of new treatment strategies.

## Data Availability

The datasets generated during and analyzed during the current study are available from the corresponding author on reasonable request.

## Abbreviations

HSP: Hereditary spastic paraplegia
